# Functional tests to guide management in an adult with loss of function of type-1 angiotensin II receptor

**DOI:** 10.1007/s00467-021-05018-7

**Published:** 2021-03-25

**Authors:** Daan H. H. M. Viering, Anneke P. Bech, Jeroen H. F. de Baaij, Eric J. Steenbergen, A. H. Jan Danser, Jack F. M. Wetzels, René J. M. Bindels, Jaap Deinum

**Affiliations:** 1 Department of Physiology, Radboud University Medical Centre, Radboud Institute for Molecular Life Sciences, Nijmegen, the Netherlands; 2 Department of Nephrology, Rijnstate, Arnhem, the Netherlands; 3 Department of Pathology, Radboud University Medical Centre, Nijmegen, the Netherlands; 4 Department of Internal Medicine, Erasmus Medical Centre, Rotterdam, the Netherlands; 5 Department of Nephrology, Radboud University Medical Centre, Nijmegen, the Netherlands; 6 Department of Internal Medicine, Radboud University Medical Centre, Huispost 463, Geert Grooteplein 8, 6525 GA Nijmegen, the Netherlands

**Keywords:** Renin-angiotensin system, Renal tubular dysgenesis, Angiotensin II receptor type 1, AGTR1, Cortical collecting duct

## Abstract

**Background:**

Genetic loss of function of *AGT* (angiotensinogen), *REN* (renin), *ACE* (angiotensin-converting enzyme), or *AGTR1* (type-1 angiotensin II receptor) leads to renal tubular dysgenesis (RTD). This syndrome is almost invariably lethal. Most surviving patients reach stage 5 chronic kidney disease at a young age.

**Methods:**

Here, we report a 28-year-old male with a homozygous truncating mutation in *AGTR1* (p.Arg216*), who survived the perinatal period with a mildly impaired kidney function. In contrast to classic RTD, kidney biopsy showed proximal tubules that were mostly normal. During the subsequent three decades, we observed evidence of both tubular dysfunction (hyperkalemia, metabolic acidosis, salt-wasting and a urinary concentrating defect) and glomerular dysfunction (reduced glomerular filtration rate, currently ~30 mL/min/1.73 m^2^, accompanied by proteinuria). To investigate the recurrent and severe hyperkalemia, we performed a patient-tailored functional test and showed that high doses of fludrocortisone induced renal potassium excretion by 155%. Furthermore, fludrocortisone lowered renal sodium excretion by 39%, which would have a mitigating effect on salt-wasting. In addition, urinary pH decreased in response to fludrocortisone. Opposite effects on urinary potassium and pH occurred with administration of amiloride, further supporting the notion that a collecting duct is present and able to react to fludrocortisone.

**Conclusions:**

This report provides living proof that even truncating loss-of-function mutations in *AGTR1* are compatible with life and relatively good GFR and provides evidence for the prescription of fludrocortisone to treat hyperkalemia and salt-wasting in such patients.

**Supplementary Information:**

The online version contains supplementary material available at 10.1007/s00467-021-05018-7.

## Introduction

Inhibitors of the renin-angiotensin system (RAS) play an important role in the management of increased cardiovascular and renal risk in the aging population. During fetal development, the RAS regulates kidney perfusion and affects kidney development [1, 2]. Children with genetic loss-of-function variants in any of the RAS components develop renal tubular dysgenesis (RTD). This clinical syndrome is characterized by poor development of especially proximal tubules, early onset and persistent anuria (often manifesting prenatally) and ossification defects of the skull. Children with pathogenic variants in a RAS component typically die *in utero* or in the first days of life and develop stage 5 chronic kidney disease (CKD 5) at a young age [3, 4]. Salt-wasting and hyperkalemia have been reported in several cases [5–7]. Biallelic pathogenic variants have been described in four essential genes of the RAS: angiotensinogen (*AGT*), renin (*REN*), angiotensin-converting enzyme (*ACE*) and the angiotensin II receptor (*AGTR1*) [3, 4]. Similarly, the use of RAS inhibitors during the second or third trimester of pregnancy can also result in RTD, further stressing the importance of the RAS for normal kidney development [8, 9].

Survival without the need for kidney replacement therapy has been described in patients with biallelic pathogenic variants in *ACE*, *AGT* or *REN* [4–7, 10–14]. Nevertheless, several questions remain unanswered. Are full loss-of-function variants in *AGTR1*, the gene encoding the most important receptor for angiotensin II, compatible with kidney survival? And how should hyperkalemia, salt-wasting and hypotension be treated in a patient with loss of *AGTR1* function? Here, we describe the case of a 28-year-old patient with homozygous pathogenic variants in *AGTR1* that provides new insights on these questions.

## Case description

**Fig. 1:**
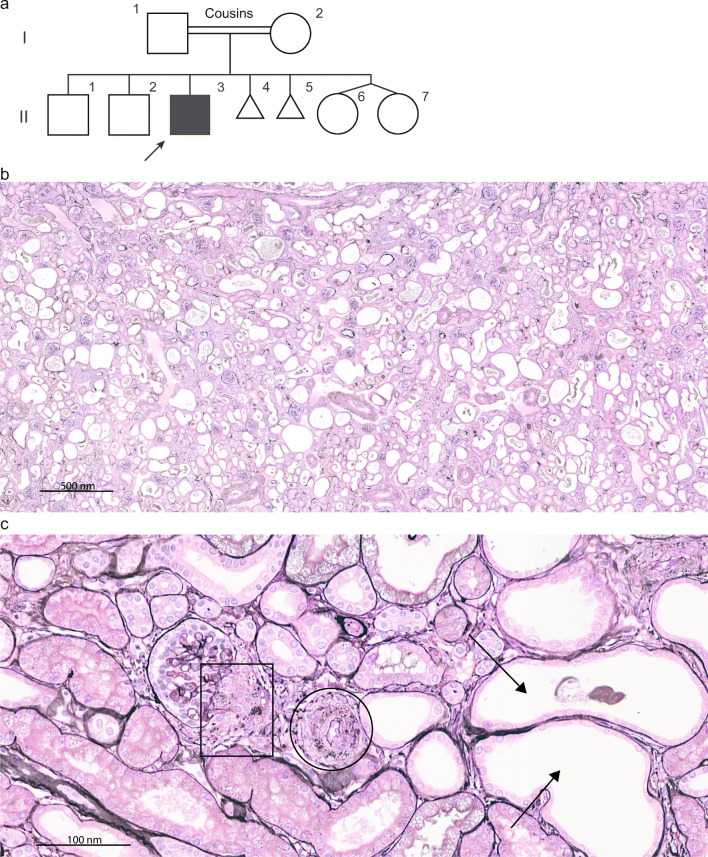
Pedigree and kidney histology of the proband. **a** Pedigree of the affected family. I.1 and I.2 are first-degree cousins. **b** and **c** Silver staining, magnification 50× and 400×. Histology of kidney of proband, obtained at age 3 months. The kidney biopsy shows microcystic changes of tubuli, mostly of distal tubules (arrows). Glomeruli show hypertrophic arterioles (circle) and well-developed juxtaglomerular apparatus (square) in which silver-stained granules are visible that may represent renin. Changes are not typical for renal tubular dysgenesis that is rather characterized by atrophic tubules. However, the prominent smooth muscle cells and renin overexpression in the juxtaglomerular apparatus is a common feature in renal tubular dysgenesis

The proband, the third son of consanguinous parents of Turkish decent (pedigree in Fig. 1a), came to our attention at the age of 18 years. The pregnancy had been complicated by oligohydramnios, and he was born at 34^+1^ weeks’ gestation by cesarean section that was urged by decreased variability in fetal heart rate. Apgar scores were 6, 8 and 9 after 1, 5 and 10 min, respectively, and birthweight was 2290 g. He was slightly hypotonic; had large ears, wide fontanelles and hypotelorism; and required oxygen therapy for a short period of time. Ten days post-partum, he developed feeding difficulties and oliguria and was noted to have hypotension (46/25 mmHg), severe kidney failure (serum creatinine 335 μmol/L), hyponatremia (118 mmol/L), hyperkalemia (up to 7 mmol/L), metabolic acidosis (7.29), a low transtubular potassium gradient of 3.9 and a mild transient glucosuria (6 mmol/L) with mild transient aminoaciduria. He was diagnosed with a salt-losing tubulopathy and responded well to intravenous sodium (Na^+^) suppleting therapy and potassium (K^+^)-binding resins although his blood pressure remained low. In his first year, he depended on gastric tube feeding with Na^+^ suppletion and underwent repair of an inguinal and umbilical hernia. Postoperatively, he developed convulsions with signs of occipital infarction, probably due to severe hypotension. Kidney ultrasounds in the first months of life showed reduced cortico-medullary differentiation, and a kidney biopsy at the age of 3 months revealed microcystic dilation of tubules with interstitial changes (Fig. 1). No further investigations were done. In his first year, at least three episodes of acute kidney injury with serum creatinine levels > 250 μmol/L were documented, including the episode shortly after birth.

Pubertal development was normal, but growth lagged behind. He received growth hormone treatment from 11 to 15 years of age and reached an adult height of 178 cm. Cognitive development initially lagged behind that of his peers, but eventually, he completed vocational education. His treating pediatrician noticed that at the age of 10 years, he had unusually elevated plasma renin levels (12,000 mU/L, normal < 75 mU/L) and inappropriately low plasma aldosterone levels (0.03 nmol/L, normal > 0.16 nmol/L). Pathogenic variants in aldosterone synthase (*CYP11B2*) were ruled out by Sanger sequencing, as were other forms of adrenogenital syndrome. The response of adrenal steroids to cosyntropin stimulation was normal. No further investigations were performed. He was polyuric, which was accompanied by enuresis nocturna until the age of 13. A desmopressin challenge showed that urinary concentrating ability was impaired (maximal urine osmolality 241 mosmol/L). He continued his sodium chloride supplementation, and fludrocortisone was added at large dosages (up to 0.3 mg/day) but with little effect on urinary salt loss. At the age of 18, when his estimated GFR (eGFR) was ~45 mL/min/1.73 m^2^, he started to develop proteinuria (0.18 g/L). Further medical history was uneventful, except for the chronic presence of cutis verticis gyrata. Also, during childhood, the patient suffered from transient recurrent anemia (4.6–5.7 mmol/L, treated with erythropoietin).

Currently, at the age of 28 years, his blood pressure is still low (systolic blood pressure around 90–100 mmHg) with an asymptomatic orthostatic increase of heart rate with 20–30 beats per minute. Creatinine levels are stable around 200 μmol/L (estimated glomerular filtration rate, eGFR ~30 mL/min/1.73 m^2^), inversely related to his salt intake. Proteinuria is persistent (protein–creatinine ratio of around 100 mg/mmol) and mainly consists of albumin (96% in last measurement). Despite sodium chloride suppletion and fludrocortisone therapy, his plasma renin levels remain elevated (between 400 and 2000 mU/L). The only other medication that he currently receives is 1-hydroxycholecalciferol.

## Methods

### Measurement of RAS components

Blood samples were taken in the seated position after 5 min of rest. Plasma renin concentration was measured by a standard enzyme-kinetic assay using sheep renin substrate [15], and plasma renin acitivity was measured according to previously described protocols [16]. Plasma angiotensinogen was measured by angiotensin I content [17]. Angiotensin peptides were measured after semipurification, HPLC-separation and radioimmunoassay [16]. At the moment of measurements, the patient was taking NaCl supplements of up to 9 g/day and fludrocortisone of 0.3 mg/day.

### Exome sequencing

Full description can be found in the Supplementary Methods. In short, genomic DNA was isolated from whole blood, converted into an Illumina library, enriched for exonic regions and sequenced on an Illumina HiSeq2000 platform. Sequence reads were aligned to Human Genome Reference Assembly GCRh37/hg19, indexed and subsequently called. Variant annotation and de novo analysis were performed using a custom-designed in-house analysis pipeline. Filtering was performed as described in Supplementary Table 1, and pathogenicity of candidate variants was assessed using the Association for Clinical Genomic Science (ACGS) Best Practice Guidelines for Variant Classification 2019. Additionally, we looked for possible pathogenic variants in *CYP11B2* (enoding aldosterone synthase) and the RAS genes in the unfiltered data.

### Collecting duct function

To assess whether collecting duct function was present and amenable to treatment with fludrocortisone, we assessed the response to amiloride and fludrocortisone. Amiloride directly blocks the epithelial Na^+^ channel (ENaC), the channel involved in Na^+^ reabsorption in the collecting duct, while fludrocortisone upregulates ENaC through its action on the mineralocorticoid receptor [18, 19]. Two days before starting the test, maintenance dose of fludrocortisone (0.3 mg/day) was stopped. The night before the first test day, the patient received 1 L NaCl 0.9% (w/v) intravenously. At 8 AM, intravenous infusion of NaCl 0.9% 50 mL/h was started, and urine was collected for measurement of sodium, potassium, creatinine and pH. At 10 AM (*t* = 0), a urine portion was collected, and directly after, amiloride of 20 mg was administered orally. Urine was collected again hourly from *t* = 120 min until *t* = 360 min for the same measurements. At *t* = 480 min, intravenous NaCl 0.9% infusion was stopped. The second day of the test was exactly the same, except that fludrocortisone of 1 mg was administered orally instead of amiloride. The only other difference was that the time of drug administration was 3 h after the start of intravenous NaCl supplementation instead of 2 h.

## Results

### Histology, plasma measurements

Renal tubular dysgenesis is normally characterized by atrophic tubules, especially seen as an extensive reduction in differentiated proximal tubules. Here, a kidney biopsy at 3 months of age showed the presence of both distal and proximal tubules, although some distal tubules had microcystic changes (Fig. 1b). Arteries were not evidently abnormal in this biopsy; however, arteriolar hypertrophy was observed.

Measurements of the RAS components in the proband and family members can be found in Table 1. In the proband, angiotensinogen was significantly reduced (737 nmol/L), but immunoreactive renin and renin activity were elevated (410 mU/L and 14.4 nmol Ang I/L·h, respectively), as were levels of angiotensin I (234 ng/L) and angiotensin II (78.2 ng/L). ACE activity was normal (17.8 U/L). Although reference ranges for healthy individuals are not readily available for all RAS components, RAS levels were not significantly abnormal in family members when compared with the reference data available [20–23]. Aldosterone levels fluctuated between abnormally low and low normal in the patient (< 0.03–0.15 nmol/L).

**Table 1:** Components of renin-angotensin-aldosterone system

	PRA	Renin	Aog	Ang I	Ang II	Aldosterone	ACE
	nmol Ang I/L·h	mU/L	nmol/L	ng/L	ng/L	nmol/L	U/L
Reference	1.2 (0.5–1.7) [20]	23 (3–116) [20]	745–2340 [21]	44 (12–182) [22]	1–20 [22, 23]	0.04–0.66	< 20
Father	1.43	28	1709	18.3	6.2	NA	
Mother	1.05	9.9	1938	BDL	0.85	NA	
Sibling 1	3.53	62.2	1529	15.5	11.7	NA	
Sibling 2	0.90	18.1	1528	7.3	1.8	NA	
Index	14.4	1900	737	234	78.2	0.13	17.8

Components of renin-angotensin-aldosterone system in family members. Aldosterone was measured in the seated position after 5 min of rest. At the moment of measurement of renin and aldosterone, the index was taking NaCl supplements of up to 3 g/day and fludrocortisone of 0.3 mg/day, and all tested individuals were of adult age

*ACE*, angiotensin-converting enzyme; *Ang*, angiotensin; *Aog*, angiotensinogen; *BDL*, below detection limit; *NA*, not assessed; *PRA*, plasma renin activity

### Detection of homozygous AGTR1 p.Arg126* variant

The low aldosterone levels despite high angiotensin II levels suggests that the patient had a defect of the angiotensin II receptor. Indeed, exome sequencing revealed a homozygous pathogenic variant in *AGTR1,* c.822C>T (NM_031850.3, dbSNP ID rs397514687), resulting in a premature stop codon (p.Arg164*, full-length AGTR1 has 395 amino acids). The location of the variant is retained in all known splice isoforms [24]. The variant is very rare; it was absent from the Exome Variant Server and has a minor allele frequency of 1.64e-5 in GnomAD, with no homozygote occurrences. Parents were heterozygous. The same variant has been reported in the homozygous state in a Pakistani family with RTD [4]. A founder effect could not be confirmed or excluded. The variant was classified as pathogenic with the ACGS 2019 guidelines.

Of note, no rare variants (minor allele frequency < 0.005) in *CYP11B2*, *REN*, *ACE* and *AGT* were found. An overview of the filtering strategy and a list of variants that remained after filtering can be found in Supplmentary Tables 1, 2 and 3.

### Functioning collecting duct

**Fig. 2:**
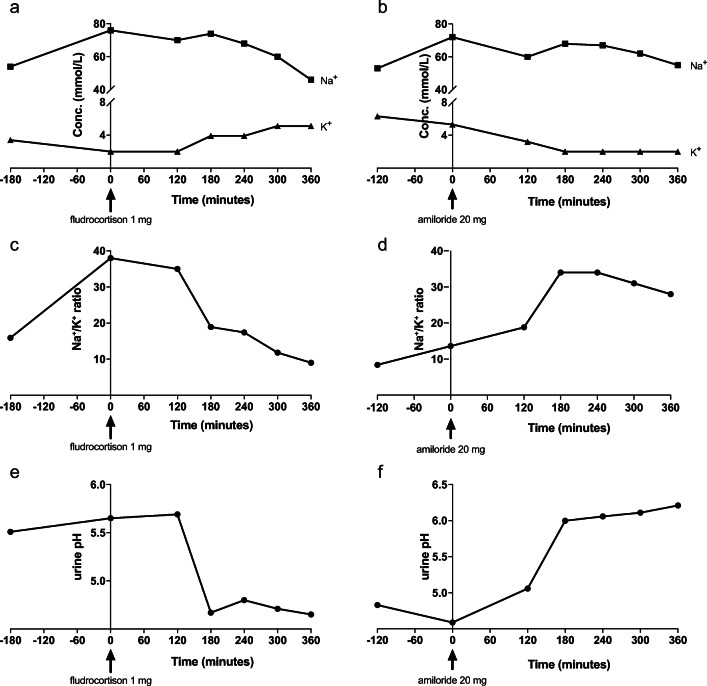
Collecting duct function in the proband. Urinary excretion of Na^+^, K^+^ and H^+^ after oral administration of amiloride 20 mg (panels **a**, **c** and **e**) or fludrocortisone 1 mg (panels **b**, **d**, and **f**) in the proband. Intravenous infusion with 0.9% (w/v) NaCl (50 mL/h) was started at the first measurement point (i.e., *t* = −120 min for panels **a**, **c**, and **e** and *t* = −180 min for panels **b**, **d** and **f**). **a** and **b** Urinary excretion of K^+^ and Na^+^. **c** and **d** Na^+^/K^+^-ratio. **e** and **f** Urinary pH

The recurrent hyperkalemia incited us to study the effect of fludrocortisone on collecting duct function. Within 3 h of administration, 1 mg fludrocortisone increased urinary K^+^ excretion to 255% of baseline and decreased Na^+^ excretion by 39%, resulting in a maximum 4.2-fold change decrease in urine Na^+^/K^+^-ratio (Fig. 2). To confirm the functionality of the collecting duct, we also measured the response to amiloride (20 mg). A decrease in urinary K^+^ excretion was observed and resulted in an increase of the urine Na^+^/K^+^-ratio by 1.8-fold. Furthermore, urinary pH decreased by 1 and increased by 1.6 after administration of fludrocortistone and amiloride, respectively.

## Discussion

In this report we describe a 28-year-old male with a homozygous truncating variant in *AGTR1* (p.Arg126*) who survived the perinatal period without the need for kidney replacement therapy. The patient reported here had a glomerular filtration rate that was relatively well preserved, especially considering the severity of the mutation and the fact that the variant affected the type-1 angiotensin II receptor, AGTR1 [4, 11]. However, other signs of kidney dysfunction affected the patient throughout life, including several episodes of acute kidney injury at young age, hypotensive episodes, chronic tubular salt-wasting, impaired urinary concentrating ability with polyuria-polydipsia, recurrent hyperkalemia, a severely reduced GFR and proteinuria. Anemia, although of transient nature, was present as well, resembling three cases with pathogenic variants in *ACE* [11]. Lastly, we provide new evidence to support the use of fludrocortisone for the treatment of recurrent hyperkalemias in RTD.

With a patient-tailored functional test, we show that fludrocortisone can increase K^+^ excretion and might mitigate Na^+^ wasting in RTD. Fludrocortisone stimulates the aldosterone receptor, resulting in ENaC upregulation and subsequent excretion of K^+^ and H^+^ ions in healthy individuals. We provide evidence that this mechanism is still operative in patients without *AGTR1* function and low levels of circulating aldosterone. In support of this mechanism, we show that H^+^ excretion is also stimulated by the administration of fludrocortisone and that amiloride has the exact opposite effect on urinary K^+^ and H^+^ excretion. The use of fludrocortisone as a treatment in RTD patients has been suggested before [6, 10, 25]. However, the exact effects of fetal RAS dysfunction on the development of the different tubular segments are still largely unknown, as is the functionality of the different segments and molecular transporters. Tests such as those we describe here are therefore of great importance to justify the lifelong use of drugs. Based on our results, fludrocortisone treatment was continued to prevent the recurrence of hyperkalemias that sometimes reached symptomatic, life-threatening levels.

The fact that our patient survived the perinatal period is remarkable. In 2014, when approximately 150 cases of RTD had been reported, data on only ten long-term survivors was available (reviewed in [1]) [1, 4, 7, 14, 26–28]. Since then, only seven more survivors have been described [5, 6, 10–12]. The patient we present here is the only reported long-term survivor with bi-allelic variants in *AGTR1*. Furthermore, most survivors had missense variants or single amino acid deletions, leaving the possibility open that some residual function was present. It has been suggested that vasopressin might be a life-saving therapy to treat hypotension in these children [6, 10, 12]. Unfortunately, most children die *in utero* or shortly after birth and thus do not get the chance to receive such treatment. As an example, two affected siblings in a Pakistani family with the same biallelic p.Arg126* *AGTR1* variant died in the first day after birth [4]. The degree of oligohydramnios in our patient was milder than what has been reported for most patients, especially judged by the extent of neonatal respiratory distress that is often observed [5, 6, 10, 12–14, 28, 29]. Furthermore, the patient reported here was not anuric or severely oligouric after birth. Lastly, the kidney biopsy did not show full-blown RTD: proximal tubules were mostly normal, in agreement with other available kidney histology reports from survivors [5, 11, 27], and although arterioles showed hypertrophic changes, arteries did not show evident wall thickening, in contrast to reports from both survivors and non-survivors [3–5, 11, 14, 30]. We hypothesize that the perinatal preservation of glomerular filtration and urine production might have protected our patient from severe complications such as pulmonary hypoplasia and the damaging effects of kidney failure itself, which, together with adequate supportive care, allowed our patient to survive the perinatal period.

Taken together, based on the findings in our patient with a homozygous truncating variant in *AGTR1*, we encourage clinicians and patients to be aware of the multitude of types of kidney dysfunction that can occur over a lifetime in survivors with RTD. Special care should be taken to avoid eliciting acute kidney injury and associated decline in glomerular filtration, complications of (peri-operative) hypotension and situations that provoke hyperkalemia. On the other hand, this study shows that the prognosis can be good, even in genetically very severe cases. Lastly, this study provides physiological evidence to support the use of fludrocortisone for hyperkalemia and salt-wasting in RTD.

## Supplementary Information

ESM 1 (PDF 190 kb)

ESM 2 (XLSX 86 kb)

## References

[1] Gubler MC (2014). Renal tubular dysgenesis. Pediatr Nephrol.

[2] Yosypiv IV (2020). Renin-angiotensin system in mammalian kidney development. Pediatr Nephrol.

[3] Gribouval O, Gonzales M, Neuhaus T, Aziza J, Bieth E, Laurent N, Bouton JM, Feuillet F, Makni S, Amar HB, Laube G, Delezoide A-L, Bouvier R, Dijoud F, Ollagnon-Roman E, Roume J, Joubert M, Antignac C, Gubler MC (2005). Mutations in genes in the renin-angiotensin system are associated with autosomal recessive renal tubular dysgenesis. Nat Genet.

[4] Gribouval O, Morinière V, Pawtowski A, Arrondel C, Sallinen S-LL, Saloranta C, Clericuzio C, Viot G, Tantau J, Blesson S, Cloarec S, Machet MC, Chitayat D, Thauvin C, Laurent N, Sampson JR, Bernstein JA, Clemenson A, Prieur F, Daniel L, Levy-Mozziconacci A, Lachlan K, Alessandri JL, Cartault F, Rivière JP, Picard N, Baumann C, Delezoide AL, Belar Ortega M, Chassaing N, Labrune P, Yu S, Firth H, Wellesley D, Bitzan M, Alfares A, Braverman N, Krogh L, Tolmie J, Gaspar H, Doray B, Majore S, Bonneau D, Triau S, Loirat C, David A, Bartholdi D, Peleg A, Brackman D, Stone R, DeBerardinis R, Corvol P, Michaud A, Antignac C, Gubler MC, Ortega MB, Chassaing N, Labrune P, Yu S, Firth H, Wellesley D, Bitzan M, Alfares A, Braverman N, Krogh L, Tolmie J, Gaspar H, Doray B, Majore S, Bonneau D, Triau S, Loirat C, David A, Bartholdi D, Peleg A, Brackman D, Stone R, DeBerardinis R, Corvol P, Michaud A, Antignac C, Gubler MC (2012). Spectrum of mutations in the renin-angiotensin system genes in autosomal recessive renal tubular dysgenesis. Hum Mutat.

[5] Hibino S, Sasaki H, Abe Y, Hojo A, Uematsu M, Sekine T, Itabashi K (2015). Renal function in angiotensinogen gene-mutated renal tubular dysgenesis with glomerular cysts. Pediatr Nephrol.

[6] Richer J, Daoud H, Geier P, Jarinova O, Carson N, Feberova J, Ben Fadel N, Unrau J, Bareke E, Khatchadourian K, Bulman DE, Majewski J, Boycott KM, Dyment DA (2015). Resolution of refractory hypotension and anuria in a premature newborn with loss-of-function of ACE. Am J Med Genet A.

[7] Schreiber R, Gubler MC, Gribouval O, Shalev H, Landau D (2010). Inherited renal tubular dysgenesis may not be universally fatal. Pediatr Nephrol.

[8] Pryde PG, Sedman AB, Nugent CE, Barr M (1993). Angiotensin-converting enzyme inhibitor fetopathy. J Am Soc Nephrol.

[9] Martinovic J, Benachi A, Laurent N, Daikha-Dahmane F, Gubler MC (2001). Fetal toxic effects and angiotensin-II-receptor antagonists. Lancet.

[10] Ruf K, Wirbelauer J, Beissert A, Frieauff E (2018). Successful treatment of severe arterial hypotension and anuria in a preterm infant with renal tubular dysgenesis- a case report. Matern Health Neonatol Perinatol.

[11] Fila M, Morinière V, Eckart P, Terzic J, Gubler MC, Antignac C, Heidet L (2020). Bi-allelic mutations in renin-angiotensin system genes, associated with renal tubular dysgenesis, can also present as a progressive chronic kidney disease. Pediatr Nephrol.

[12] Kondoh T, Kawai Y, Matsumoto Y, Kumagai N, Miyata M, Tanaka K, Hibino S, Fujita N, Ikezumi Y (2020). Management of a preterm infant with renal tubular dysgenesis: a case report and review of the literature. Tohoku J Exp Med.

[13] Kim SY, Kang HG, Kim EK, Choi JH, Choi Y, Cheong HI (2012). Survival over 2 years of autosomal-recessive renal tubular dysgenesis. Clin Kidney J.

[14] Uematsu M, Sakamoto O, Nishio T, Ohura T, Matsuda T, Inagaki T, Abe T, Okamura K, Kondo Y, Tsuchiya S (2006). A case surviving for over a year of renal tubular dysgenesis with compound heterozygous angiotensinogen gene mutations. Am J Med Genet A.

[15] Derkx FH, Tan-Tjiong L, Wenting GJ, Boomsma F, Man in 't Veld AJ, Schalekamp MA (1983). Asynchronous changes in prorenin and renin secretion after captopril in patients with renal artery stenosis. Hypertension.

[16] Admiraal P, Danser A, Jong MS, Pieterman H, Derkx F, Schalekamp M (1993). Regional angiotensin II production in essential hypertension and renal artery stenosis. Hypertension.

[17] Danser AJ, van Kesteren CA, Bax WA, Tavenier M, Derkx FH, Saxena PR, Schalekamp MA (1997). Prorenin, renin, angiotensinogen, and angiotensin-converting enzyme in normal and failing human hearts: evidence for renin binding. Circulation.

[18] Kellenberger S, Schild L (2015). International Union of Basic and Clinical Pharmacology. XCI. Structure, function, and pharmacology of acid-sensing ion channels and the epithelial Na+ channel. Pharmacol Rev.

[19] Walsh SB, Shirley DG, Wrong OM, Unwin RJ (2007). Urinary acidification assessed by simultaneous furosemide and fludrocortisone treatment: an alternative to ammonium chloride. Kidney Int.

[20] Campbell DJ, Nussberger J, Stowasser M, Danser AH, Morganti A, Frandsen E, Ménard J (2009). Activity assays and immunoassays for plasma renin and prorenin: information provided and precautions necessary for accurate measurement. Clin Chem.

[21] Derkx FH, Stuenkel C, Schalekamp MP, Visser W, Huisveld IH, Schalekamp MA (1986). Immunoreactive renin, prorenin, and enzymatically active renin in plasma during pregnancy and in women taking oral contraceptives. J Clin Endocrinol Metab.

[22] Admiraal PJ, Derkx FH, Danser AH, Pieterman H, Schalekamp MA (1990). Metabolism and production of angiotensin I in different vascular beds in subjects with hypertension. Hypertension.

[23] Balcarek J, Sevá Pessôa B, Bryson C, Azizi M, Ménard J, Garrelds IM, McGeehan G, Reeves RA, Griffith SG, Danser AH, Gregg R (2014). Multiple ascending dose study with the new renin inhibitor VTP-27999: nephrocentric consequences of too much renin inhibition. Hypertension.

[24] Curnow KM, Pascoe L, Davies E, White PC, Corvol P, Clauser E (1995). Alternatively spliced human type 1 angiotensin II receptor mRNAs are translated at different efficiencies and encode two receptor isoforms. Mol Endocrinol.

[25] Min J, Cho MH, Bae SP, Shin SH, Ha IS, Cheong HI, Kang HG (2020). A premature baby with severe oligohydramnios and hypotension: a case report of renal tubular dysgenesis. J Korean Med Sci.

[26] Bacchetta J, Dijoud F, Bouvier R, Putet G, Gubler MC, Cochat P (2007). Renal tubular dysgenesis and mutation in the renin gene. Arch Pediatr.

[27] Zingg-Schenk A, Bacchetta J, Corvol P, Michaud A, Stallmach T, Cochat P, Gribouval O, Gubler MC, Neuhaus TJ (2008). Inherited renal tubular dysgenesis: the first patients surviving the neonatal period. Eur J Pediatr.

[28] Uematsu M, Sakamoto O, Ohura T, Shimizu N, Satomura K, Tsuchiya S (2009). A further case of renal tubular dysgenesis surviving the neonatal period. Eur J Pediatr.

[29] Dilliott AA, Wang J, Brown E, Singh G, Shkrum MJ, Clin M, Rupar CA, Hegele RA, Siu VM (2020). A novel homozygous variant in REN in a family presenting with classic features of disorders involving the renin-angiotensin pathway, without renal tubular dysgenesis. Am J Med Genet A.

[30] Gubler MC, Antignac C (2010). Renin-angiotensin system in kidney development: renal tubular dysgenesis. Kidney Int.

