# Supplementary material for: Functional tests to guide management in an adult with loss of function of type-1 angiotensin II receptor
Source: Pediatr Nephrol. 2021 Mar 25;36(9):2731–7. doi: 10.1007/s00467-021-05018-7 (PMC8370907; doi:10.1007/s00467-021-05018-7)
Supplement: Supplementary file 1 — (PDF 190 kb) [file 467_2021_5018_MOESM1_ESM.pdf]

Functional tests to guide management in an adult with loss of function of type-1 angiotensin II receptor

Supplementary material

Daan HHM Viering[1], MD; Anneke P Bech[2], MD, PhD; Jeroen HF de Baaij[1], PhD; Eric J Steenbergen[3], MD, PhD; prof. AH Jan Danser[4], PhD; prof. Jack FM Wetzels[5], MD, PhD; prof. René JM Bindels[1], PhD; Jaap Deinum[6], MD, PhD.

Affilliations:
1. Radboud university medical centre, Radboud Institute for Molecular Life Sciences, Department of Physiology, Nijmegen, the Netherlands
2. Rijnstate, Department of Nephrology, Arnhem, the Netherlands
3. Radboud university medical centre, Department of Pathology, Nijmegen, the Netherlands
4. Erasmus Medical Centre, Department of Internal Medicine, Rotterdam, the Netherlands
5. Radboud university medical centre, Department of Nephrology, Nijmegen, the Netherlands
6. Radboud university medical centre, Department of Internal Medicine, Nijmegen, the Netherlands

Corresponding author
Jaap Deinum
Department of Internal Medicine, Huispost 463
Radboud university medical center
Geert Grooteplein 8, 6525 GA Nijmegen
Email: jaap.deinum@radboudumc.nl

Supplementary methods

DNA preparation and next-generation sequencing

The original samples were submitted to Otogenetics Corporation (Atlanta, GA USA) for human exome capture and sequencing. Genomic DNA (gDNA) was isolated from whole blood using the FlexiGene DNA Kit (cat. 51204, Qiagen). Briefly, gDNA was subjected to agarose gel and OD ratio tests via Nanodrop to confirm the purity and concentration prior to Bioruptor (Diagenode, Inc., Denville, NJ USA) fragmentation. Fragmented gDNAs were tested for size distribution and concentration using an Agilent Tapestation 2200. Illumina libraries were made from qualified fragmented gDNA using New England Biolabs DNA Library Prep Master Mix Set for Illumina (New England Biolabs. Ipswich, MA USA, catalog# 6040L) and the resulting libraries were subjected to exome enrichment using SureSelectXT human All Exon V4 (Agilent Technologies, Wilmington, DE USA, catalog# 5190-4641) following manufacturer's instructions. Enriched libraries were tested for enrichment by qPCR and for size distribution and concentration by an Agilent Bioanalyzer 2100. The samples were then sequenced on an Illumina HiSeq2000 which generated paired-end reads of 100 nucleotides (nt). Data was analyzed for data quality using FASTQC (Babraham Institute, Cambridge, UK).

Bioinformatics Analysis and Pipeline

Sequence reads were aligned to the Human Genome Reference Assembly GCRh37/hg19 using Burrows-Wheeller Alignment (BWA) version 0.7.12,[1] and indexed using SAMtools version 1.6.[2] SNVs and indels were subsequently called by the Genome Analysis Toolkit (GATK) HaplotypeCaller version 3.4-46. Variant annotation and *de novo* analysis were performed using a custom-designed in-house analysis pipeline.

Genotypes were compared to population frequencies as reported by gnomAD v2.1.1, the EVS data release ESP6500SI-V2 (NHLBI GO ESP Exome Variant Server) and an in-house database. Filtering was done with the filtering steps described in Supplementary table 1. Pathogenicity of the variant was assessed using the Association for Clinical Genomic Science (ACGS) Best Practice Guidelines for Variant Classification 2019 (https://www.acgs.uk.com/quality/best-practice-guidelines/, ratified 06 May 2019).

To confirm or exclude a genetic defect in the renin-angiotensin-aldosterone system, we also looked specifically for any rare variants (i.e. < 0.005 minor allele frequency) in *CYP11B2*, *REN*, *ACE*, *AGT* and *AGTR1* in the unfiltered data.

References

1 Li, H. & Durbin, R. Fast and accurate long-read alignment with Burrows–Wheeler transform. *Bioinformatics* **26**, 589-595, doi:10.1093/bioinformatics/btp698 (2010).

2 Li, H. *et al.* The Sequence Alignment/Map format and SAMtools. *Bioinformatics* **25**, 2078-2079, doi:10.1093/bioinformatics/btp352 (2009).

Supplementary tables

Supplementary Table 1. Filtering strategy

This table describes the filtering steps taken after obtaining the exome sequencing data, as well as the number of variants left at each step. After filtering steps 1-3, we took two different approaches to look for causative variants, resulting in Supplementary Tables 2 and 3. We also looked for homozygous and compound heterozygous variants, resulting in the variants listed in Supplementary 3.

Step	Filter	# of variants in index	Notes
**0**	Before filtering	94282	
**1**	SNP frequency of in-house databases <0.5%	2077	
**2**	Keeping only variants in coding regions and splice sites	625	
**3**	Excluding synonymous variants	408	
			Steps 4.1 and 4.2 have been performed seperately
**4.1**	Possible de novo variants	8	Supplementary table 2
**4.2**	Keep only homozygous, hemizygous and compound heterozygous variants	28	Supplementary table 3

Supplementary Table 2. Candidate variants, de novo

After following the filtering steps described in Supplementary Table 1, we looked for possibly *de novo* pathogenic variants.

Supplementary Table 3. Candidate variants, homozygous, hemizygous and compound heterozygous

After following the filtering steps described in Supplementary Table 1, we looked for homozygous, hemizygous and compound heterozygous variants.
